# In Vitro and In Vivo Pharmaco-Toxicological Characterization of 1-Cyclohexyl-x-methoxybenzene Derivatives in Mice: Comparison with Tramadol and PCP

**DOI:** 10.3390/ijms22147659

**Published:** 2021-07-17

**Authors:** Sabrine Bilel, Micaela Tirri, Raffaella Arfè, Chiara Sturaro, Anna Fantinati, Virginia Cristofori, Tatiana Bernardi, Federica Boccuto, Marco Cavallo, Alessandro Cavalli, Fabio De-Giorgio, Girolamo Calò, Matteo Marti

**Affiliations:** 1 Section of Legal Medicine and LTTA Centre, Department of Translational Medicine, University of Ferrara, 44121 Ferrara, Italy; sabrine.bilel@unife.it (S.B.); micaela.tirri@unife.it (M.T.); raffaella.arfe@unife.it (R.A.); 2 Section of Pharmacology, Department of Neuroscience and Rehabilitation, University of Ferrara, 44121 Ferrara, Italy; chiara.sturaro@unife.it; 3 Department of Chemistry and Pharmaceutical Sciences, University of Ferrara, 44121 Ferrara, Italy; anna.fantinati@unife.it (A.F.); virginia.cristofori@unife.it (V.C.); tatiana.bernardi@unife.it (T.B.); federicaboccuto@gmail.com (F.B.); 4 NPS Section and Synthetic Drugs, Central Directorate for Anti-Drug Services (DCSA), 00173 Rome, Italy; marco.cavallo@interno.it (M.C.); cavalli.alessandro2@gdf.it (A.C.); 5 Section of Legal Medicine, Department of Health Care Surveillance and Bioethics, Università Cattolica del Sacro Cuore, 00168 Rome, Italy; 6 Fondazione Policlinico Universitario A. Gemelli IRCCS, 00168 Rome, Italy; 7 Department of Pharmaceutical and Pharmacological Sciences, University of Padua, 35122 Padua, Italy; girolamo.calo@unipd.it; 8 Collaborative Center for the Italian National Early Warning System, Department of Anti-Drug Policies, Presidency of the Council of Ministers, 00186 Roma, Italy

**Keywords:** 1-cyclohexyl-x-methoxybenzene, opioid receptors, tramadol, PCP, behavior, mice, novel psychoactive substances

## Abstract

1-cyclohexyl-x-methoxybenzene is a novel psychoactive substance (NPS), first discovered in Europe in 2012 as unknown racemic mixture of its three stereoisomers: ortho, meta and para. Each of these has structural similarities with the analgesic tramadol and the dissociative anesthetic phencyclidine. In light of these structural analogies, and based on the fact that both tramadol and phencyclidine are substances that cause toxic effects in humans, the aim of this study was to investigate the in vitro and in vivo pharmacodynamic profile of these molecules, and to compare them with those caused by tramadol and phencyclidine. In vitro studies demonstrated that tramadol, ortho, meta and para were inactive at mu, kappa and delta opioid receptors. Systemic administration of the three stereoisomers impairs sensorimotor responses, modulates spontaneous motor activity, induces modest analgesia, and alters thermoregulation and cardiorespiratory responses in the mouse in some cases, with a similar profile to that of tramadol and phencyclidine. Naloxone partially prevents only the visual sensorimotor impairments caused by three stereoisomers, without preventing other effects. The present data show that 1-cyclohexyl-x-methoxybenzene derivatives cause pharmaco-toxicological effects by activating both opioid and non-opioid mechanisms and suggest that their use could potentially lead to abuse and bodily harm.

## 1. Introduction

Over the last decade, an increasing number of new substances, known as new psychoactive substances (NPS), have been detected on the European market. The increase in the number of these substances is the result of a significant shift in the way that drugs can now be manufactured, marketed and sold, which was driven by rapid changes in both technology and globalization [1]. This rapidly changing environment has led to confusion for clinicians, psycho-pharmacologists, and the public at large [2]. In addition to the “classical” NPS, which are classified into known classes of compounds (i.e., cathinones, cannabinoids, phenethylamines, opioids, tryptamines, benzodiazepines, and dissociative anesthetics), law enforcement carries out seizures of compounds that are not classified into these groups of molecules, which are labeled as “other substances” [1].

Reviving abandoned drugs by mining old sources (e.g., from chemical journals or patents), or creating new entities with slight or major structural variations, can transform the restricted progenitor drug into an uncertain category of legal status, a “legal gray zone”. The allure of NPS is magnified by the current lack of reference materials and the need for sophisticated detection methods which are not routinely available (e.g., mass spectroscopy).

**Figure 1:**
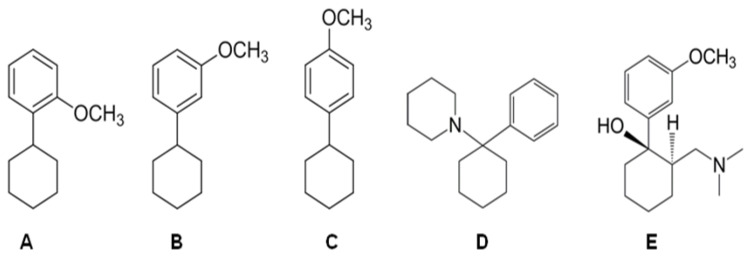
Chemical structures of (**A**) 1-cyclohexyl-2-methoxybenzene (ortho), (**B**) 1-cyclohexyl-3-methoxybenzene (meta), (**C**) 1-cyclohexyl-4-methoxybenzene (para), (**D**) phencyclidine (PCP) and (**E**) tramadol.

One case, first brought to the attention of EMCDDA and Europol in 2012, under the terms of Council Decision 2005/387/JHA, focuses on 1-cyclohexyl-x-methoxybenzene. This molecule was first identified in Austria on February 3, 2012, in white powder form. It is important to underline that the “street name” of 1-cyclohexyl-x-methoxybenzene has yet to be discovered. This is highly significant to the understanding of the potential impact of this substance on the “gray” market. The drug shares some structural analogies with tramadol and phencyclidine (PCP) (Figure 1; [3,4,5]). Therefore, these substances could be used as substitutes for tramadol and PCP to avoid clinical-toxicological and forensic identification.

In our previous study [5] we synthetized the 1-cyclohexyl-x-methoxybenzene derivatives (ortho, meta and para stereoisomers) and, for the first time, investigated some of the pharmacological effects caused by these stereoisomers. We demonstrated that systemic administration of 1-cyclohexyl-x-methoxybenzene derivatives impairs visual sensorimotor response, thermal analgesia and modulates core temperature, without affecting motor performance on the accelerod test, in CD-1 mice. Therefore, we suggested that the derivatives not only share some structural characteristics with tramadol and PCP, they also could evoke a similar pharmacological profile, thereby potentially representing a similar threat to human health [5]. Tramadol is an atypical, phenylpiperidine, centrally acting synthetic analgesic used to treat moderate to severe pain, with antinociceptive effects that are mediated by a combination of mu-opioid agonist effects and norepinephrine and serotonin reuptake inhibition [6]. Globally, the trafficking and consumption of fake tramadol has been increasing since 2007. Organized crime, armed groups and terrorist organizations were involved in the marketing of tramadol [7], as highlighted by the quantities of tramadol seized in Europe in 2017 [8].

On the other hand, phencyclidine (PCP) is a potent hallucinogenic drug, representing a synthetic arylcyclohexylamine, originally developed as an anesthetic that acts as a glutamatergic N-methyl-D-aspartate (NMDA) antagonist, also showing cholinergic and monoaminergic activity [9]. Both drugs are abused [2,10] and produce severe adverse effects, characterized by sensory changes with dissociative, out-of-body feelings and distorted visual and auditory perception. Cognitive changes, such as memory impairment, altered perception of time and slowness are common, as are affective changes, although these are quite labile, varying between euphoria, anxiety, apathy and irritability. Unpredictable changes in behavior (including aggression) and changes in consciousness are also not uncommon. Moreover, there are considerable risks associated with their use, including pulmonary edema, cerebrovascular accidents, cardiac arrest and death by overdose [2,8,11,12]. Given the large increase in the use and abuse of tramadol and the resurging interest in PCP-type substances [7], 1-cyclohexyl-x-methoxybenzene derivatives could also attract the attention of consumers and be sold in the NPS market as substitutes or alternatives to tramadol and PCP.

1-cyclohexyl-x-methoxybenzene may be sold not as a pure stereoisomer composition but as racemic mixture containing different amounts of the three ortho, meta and para stereoisomers. The 1-cyclohexyl-x-methoxybenzene compound was sequestered and identified, without determination of the composition of the racemic mixture in its three ortho, meta and para strereoisomers. This is of great relevance, as the different replacements of the methoxy group on the benzyl ring may confer different pharmacological and toxicological properties to the molecule [5]. In fact, as reported for other NPS (synthetic cannabinoids), the substitution of a hydroxyl group in the para, meta or ortho position on the benzene ring of the naphthoylindole structure causes a change in the pharmacodynamic properties and biological activity of compounds [13,14].

The aim of this study was to investigate the pharmacodynamic profile of the ortho-, meta-, and para-1-cyclohexyl-x-methoxybenzene derivatives (and tramadol for comparison) at mu, kappa and delta opioid receptors. To this aim, we used a calcium mobilization assay performed in CHO cells co-expressing human recombinant opioid receptors and chimeric G proteins that force Gi receptors to couple with the PLC-IP3-Ca2+ pathway. This assay, previously validated for the nociceptin opioid peptide receptor (NOP) [15] and later extended to classical opioid receptors [16], was used to pharmacologically characterize a large number of novel ligands, obtaining very similar results to those attained with classical assays for Gi-coupled receptors. Moreover, we investigated the effect of acute systemic administration of the single stereoisomers on neurological alterations (i.e., tail elevation, hyperreflexia and convulsions), sensorimotor responses (to visual and acoustic stimulation), body temperature, mechanical and thermal analgesia, akinesia (bar test), motor activity (spontaneous locomotion and accelerod test) and cardio-respiratory (breath rate, SpO2, hearth rate, pulse distension) changes in CD-1 male mice. In order to better characterize the pharmaco-toxicological profile of the derivatives, we compared their effects with those induced by tramadol and PCP. Moreover, to verify if 1-cyclohexyl-x-methoxybenzene derivatives have an opioid action, we studied their effect and, for comparison, that of tramadol and PCP, after administration of naloxone.

## 2. Results

### 2.1. In Vitro

In these experiments, we evaluated the ability of the tramadol and ortho-, meta-, and para-1-cyclohexyl-x-methoxybenzene derivatives to activate the mu, kappa and delta human recombinant receptors that were stably transfected in CHO cells. In such cells, the co-expression of a chimeric G protein allows for receptor activation to be measured with an automated calcium mobilization assay. Dermorphin, Dynorphin A and DPDPE were used as standard agonists for mu, kappa and delta receptors, respectively.

**Figure 2:**
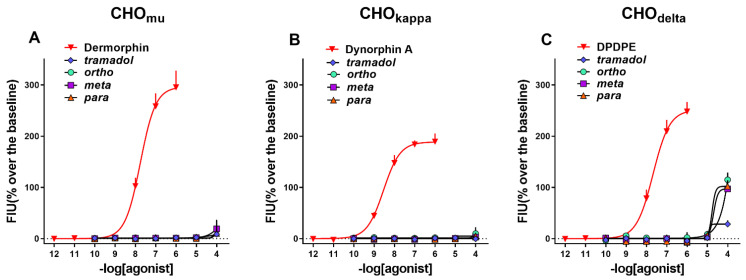
Calcium mobilization assay. Concentration response curves of standard opioid agonists, tramadol, and ortho-, meta- and para-1-cyclohexyl-X-methoxybenzene tested in CHOmu (**A**), CHOkappa (**B**), and CHOdelta cells (**C**). Data are the mean ± sem of 4 separate experiments performed in duplicate.

In CHOmu cells, the standard agonist Dermorphin evoked a robust concentration-dependent stimulation of calcium release, displaying a pEC50 of 7.76 and maximal effects of 295 ± 33% over the basal values. Tramadol and the three analogs were completely inactive. The concentration response curves obtained with these ligands are displayed in Figure 2A.

In CHOkappa cells, the standard agonist Dynorphin A evoked a robust concentration-dependent stimulation of calcium release, displaying a pEC50 of 8.50 and maximal effects of 198 ± 16% over the basal values. Tramadol and its three analogs were completely inactive. The concentration response curves obtained with these ligands are displayed in Figure 2B.

In CHOdelta cells, the standard agonist DPDPE evoked a robust concentration-dependent stimulation of calcium release, displaying high potency (pEC50 of 7.65) and maximal effects of 248 ± 19% over the basal values. Tramadol was completely inactive, while the three analogs displayed a stimulatory effect only at the higher concentration tested. The concentration response curves obtained with these ligands in CHOdelta cells are displayed in Figure 2C.

The results of these experiments are summarized in Table 1.

### 2.2. In Vivo

#### 2.2.1. Major Neurological Changes

Systemic administration of 1-cyclohexyl-x-methoxybenzene derivatives (0.1–100 mg/kg i.p.) and PCP (0.01–10 mg/kg i.p.) did not induce neurological changes, such as convulsions, hyperreflexia, myoclonia and tail elevation. Conversely, tramadol at the highest dose tested (100 mg/kg) caused tail elevation and convulsive episodes. The elevation of the tail preceded the appearance of the first seizure episode and was present in 90% of the tramadol treated animals, with a latency of appearance of 121 ± 24 s and an average duration of 65 ± 24 min. The average score was 3.5 (arbitrary units) and reached the maximum value (four arbitrary units) in approximately 78% of the treated mice. Pretreatment with naloxone 6 mg/kg did not prevent the elevation of the tail induced by tramadol at 100 mg/kg.

Tramadol induced convulsive episodes (average number of episodes 1.6 ± 0.18) in 90% of the treated animals, with a latency of 236 ± 14 s and a mean duration of 270 ± 30 s. Pretreatment with naloxone 6 mg/kg worsened the seizure effect of tramadol. It reduces the latency time of the first convulsive episode (176 ± 13 s; unpaired *t*-test: *p* = 0.0072, t = 3.141, df = 14) and increases the number of convulsive episodes (average number of episodes 2.7 ± 0.11; unpaired *t*-test: *p* = 0.0103, t = 2.964, df = 14), but did not significantly increase their overall duration (360 ± 39 s; unpaired *t*-test: *p* = 0.0888, t = 1.829, df = 14)

#### 2.2.2. Sensorimotor Studies

##### Evaluation of the Visual Object Response

**Figure 3:**
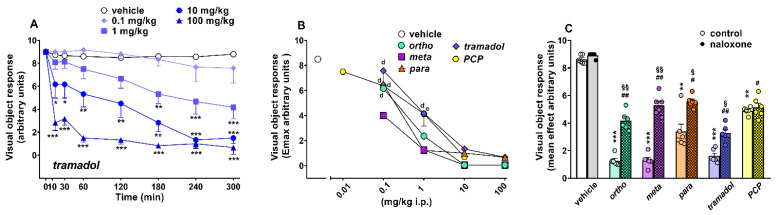
Effect of the systemic administration of tramadol (0.1–100 mg/kg i.p.; (**A**)) on the visual object test in mice. Comparison of the maximal effect of ortho, meta, para, tramadol (0.1–100 mg/kg) and PCP (0.01–10 mg/kg) observed in 5 h (**B**) ^1,2^. Interaction of the maximal effective dose of ortho, meta, para, tramadol (100 mg/kg) and PCP (10 mg/kg) with the opioid receptor antagonist naloxone (6 mg/kg, i.p.; (**C**)). Data are expressed as arbitrary units and represent the mean ± SEM of 6–8 determinations for each treatment. Statistical analysis was performed by two-way ANOVA, followed by the Bonferroni’s test for multiple comparisons for the dose–response curve of each compound at different times (**A**), and for the antagonist studies (**C**), while the statistical analysis of (**B**) was performed with one-way ANOVA followed by Bonferroni test for multiple comparisons. * *p* < 0.05, ** *p* < 0.01, *** *p* < 0.001 versus vehicle; ^d^ *p* < 0.05 versus meta; ^e^ *p* < 0.05 versus para; ^#^ *p* < 0.05, ^##^ *p* < 0.01 versus naloxone; ^§^ *p* < 0.05, ^§§^ *p*< 0.01 versus without naloxone. ^1^ ortho, meta and para data are elaborated from [5]; ^2^ PCP data are elaborated from [17].

Visual object response did not change in vehicle-treated mice over 5 h of observation (Figure 3A), and the effect was similar to that observed in naïve untreated animals (data not shown). Systemic administration of 1-cyclohexyl-x-methoxybenzene derivatives (0.1–100 mg/kg) dose-dependently inhibited visual object responses in mice. Data of 1-cyclohexyl-x-methoxybenzene derivatives (ortho, meta and para) are replicated from [5]. Systemic administration of tramadol (0.1–100 mg/kg, i.p.; Figure 3A) significantly (*p* < 0.0001) and dose-dependently reduced the visual object response in mice ((significant effect of treatment (F_4,280_ = 129.5), time (F_7,280_ = 25.43) and time x treatment interaction (F_28,280_ = 4.147)). Tramadol produced an impairment, especially at the highest dose of 100 mg/kg i.p., which reached the maximum inhibitory effect at 60 min and persisted for up to 5 h. Systemic administration of PCP (0.01–10 mg/kg) dose- dependently inhibited visual object responses in mice. PCP data are replicated from [17]. A comparison of the maximal effect among 1-cyclohexyl-x-methoxybenzene derivatives, tramadol and PCP (Figure 3B) revealed significant differences between the effects of these compounds ((significant effect of treatment (F_4,175_ = 33.09; *p* < 0.0001), dose (F_4,175_ = 103.5; *p* < 0.0001) and dose x treatment interaction (F_16,175_ = 4.28; *p* < 0.0001)). Meta at 0.1 mg/kg was the powerful compound, inducing the maximal inhibitory effect on visual object responses (*p* < 0.05). Moreover, the meta and para at 1 mg/kg were more effective with respect to tramadol and PCP for the object visual impairment (*p* < 0.05). Pretreatment with naloxone 6 mg/kg partially prevented the inhibitory effect induced by ortho, meta, para and tramadol (100 mg/kg), while the effect of PCP 10 mg/kg was naloxone-insensitive (Figure 3C).

##### Evaluation of the Visual Placing Response

**Figure 4:**
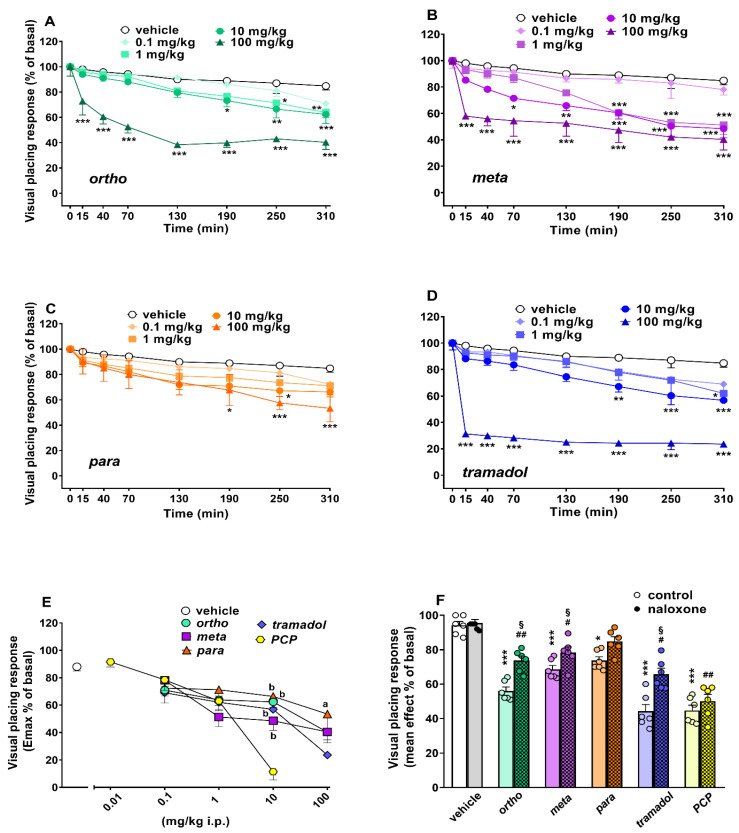
Effects of the systemic administration (0.1–100 mg/kg i.p.) of ortho (**A**), meta (**B**), para (**C**) and tramadol (**D**) on the visual placing test in mice. Comparison of the maximal effect of ortho, meta, para, tramadol (0.1–100 mg/kg) and PCP (0.01–10 mg/kg) observed in 5 h (**E**) ^2^. Interaction of the effects of ortho, meta, para, tramadol (100 mg/kg) and PCP (10 mg/kg) with the opioid receptor antagonist naloxone (6 mg/kg, i.p.; (**E**)). Data are expressed as a percentage of baseline and represent the mean ± SEM of 6–8 determinations for each treatment. Statistical analysis was performed by two-way ANOVA, followed by the Bonferroni’s test for multiple comparisons for the dose–response curve of each compound at different times (**A**–**D**), and for the antagonist studies (**F**), while the statistical analysis of (**E**) was performed with one-way ANOVA followed by Bonferroni test for multiple comparisons. * *p* < 0.05, ** *p* < 0.01, *** *p* < 0.001 versus vehicle; ^a^ *p* < 0.05 versus tramadol; ^b^ *p* < 0.05 versus PCP; ^#^ *p* < 0.05, ^##^ *p* < 0.01 versus naloxone; ^§^ *p* < 0.05 versus without naloxone. ^2^ PCP data are elaborated from [17].

Visual placing response did not change in vehicle-treated mice over 5 h of observation (Figure 4A–D), and the effect was similar to that observed in naïve untreated animals (data not shown). Systemic administration (0.1–100 mg/kg i.p.) of ortho (Figure 4A; significant effect of treatment (F_4,280_ = 105.0, *p* < 0.0001), time (F_7,280_ = 45.07, *p* < 0.0001) and time x treatment interaction (F_28,280_ = 3.433, *p* < 0.0001)), meta (Figure 4B; significant effect of treatment (F_4,280_ = 66.29, *p* < 0.0001), time (F_7,280_ = 32.83, *p* < 0.0001) and time x treatment interaction (F_28,280_ = 2.525, *p* < 0.0001)), para (Figure 4C; significant effect of treatment (F_4,280_ = 12.33, *p* < 0.0001), time (F_7,280_ = 19.13, *p* < 0.0001) but not time x treatment interaction (F_28,280_ = 0.6801, *p* = 0.8897)) and tramadol (Figure 4D; significant effect of treatment (F_4,280_ = 190.8, *p* < 0.0001), time (F_7,280_ = 38.10, *p* < 0.0001) and time x treatment interaction (F_28,280_ = 4.715, *p* < 0.0001)) reduced the visual placing response in mice in a dose-dependent manner, with the effect persisting for up to 5 h at higher doses. Systemic administration of PCP (0.01–10 mg/kg) dose-dependently inhibited visual placing responses in mice. PCP data are replicated from [17].

A comparison of the maximal effect among 1-cyclohexyl-x-methoxybenzene derivatives, tramadol and PCP (Figure 4E) revealed significant differences between the effects of these compounds ((significant effect of treatment (F_4,175_ = 3.176; *p* = 0.0156), dose (F_4,175_ = 47.92; *p* < 0.0001) and dose x treatment interaction (F_16,175_ = 3.889; *p* < 0.0001)). Notably, PCP at 10 mg/kg was the most powerful compound in inducing the maximal inhibitory effect on the visual placing responses (*p* < 0.05). Moreover, tramadol at 100 mg/kg was more effective with respect to para regarding visual placing impairment (*p* < 0.05). Pretreatment with naloxone 6 mg/kg partially prevented the inhibitory effect induced by ortho, meta and tramadol (100 mg/kg), while the effect of PCP 10 mg/kg was naloxone-insensitive (Figure 4F).

##### Evaluation of the Acoustic Response

**Figure 5:**
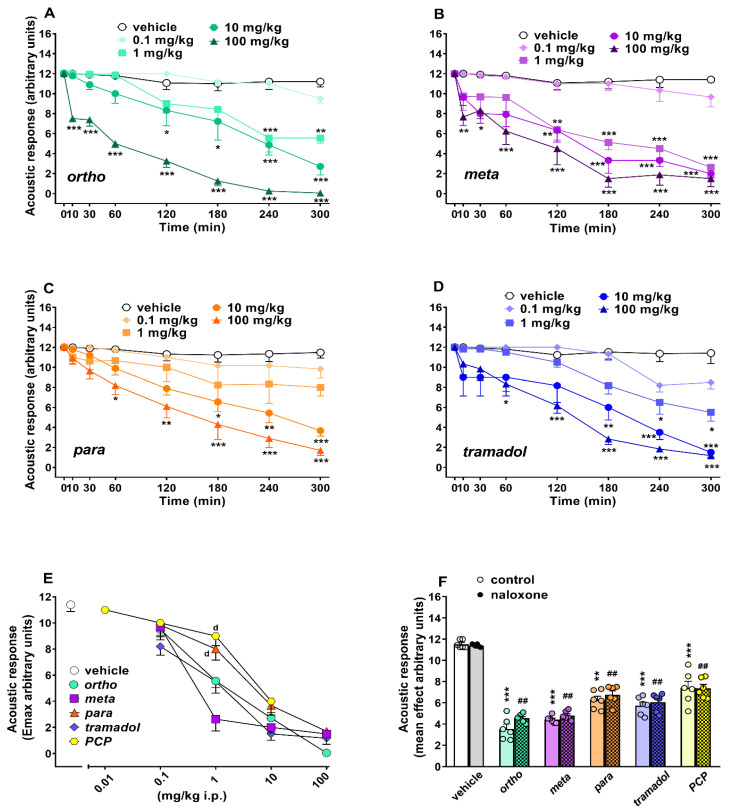
Effects of the systemic administration (0.1–100 mg/kg i.p.) of ortho (**A**), meta (**B**), para (**C**) and tramadol (**D**) on the acoustic response in mice. Comparison of the maximal effect of ortho, meta, para, tramadol (0.1–100 mg/kg) and PCP (0.01–10 mg/kg) observed over 5 h (**E**) ^2^. Interaction of the effects of ortho, meta, para, tramadol (100 mg/kg) and PCP (10 mg/kg) with the opioid receptor antagonist naloxone (6 mg/kg, i.p.; (**F**)). Data are expressed as arbitrary units and represent the mean ± SEM of 6–8 determinations for each treatment. Statistical analysis was performed by two-way ANOVA, followed by the Bonferroni’s test, for multiple comparisons of the dose–response curve of each compound at different times (**A**–**D**), and for the antagonist studies (**E**), while the statistical analysis of panel F was performed with one-way ANOVA followed by Bonferroni test for multiple comparisons. * *p* < 0.05, ** *p* < 0.01, *** *p* < 0.001 versus vehicle; ^d^ *p* < 0.05 versus meta; ^##^ *p* < 0.01 versus naloxone; ^2^ PCP data are elaborated from [17].

Acoustic response did not change in vehicle-treated mice over 5 h of observation (Figure 5A–D), and the effect was similar to that observed in naïve untreated animals (data not shown). Systemic administration (0.1–100 mg/kg i.p.) of ortho (Figure 5A; significant effect of treatment (F_4,280_ = 155.5, *p* < 0.0001), time (F_7,280_ = 60.48, *p* < 0.0001) and time × treatment interaction (F_28,280_ = 7.714, *p* < 0.0001)), meta (Figure 5B; significant effect of treatment (F_4,280_ = 85.99, *p* < 0.0001), time (F_7,280_ = 36.88, *p* < 0.0001) and time x treatment interaction (F_28,280_ = 4.026, *p* < 0.0001)), para (Figure 5C; significant effect of treatment (F_4,280_ = 36.98, *p* < 0.0001), time (F_7,280_ = 23.45, *p* < 0.0001) but not time x treatment interaction (F_28,280_ = 2.946, *p* = 0.8897)) and tramadol (Figure 5D; significant effect of treatment (F_4,280_ = 53.86, *p* < 0.0001), time (F_7,280_ = 40.41, *p* < 0.0001) and time x treatment interaction (F_28,280_ = 3.747, *p* < 0.0001)) reduced the visual placing response in mice in a dose-dependent manner, with the effect persisting for up to 5 h at higher doses.

Ortho, meta and tramadol inhibited the acoustic responses at a dose of 1 mg/kg, while the para was effective from 10 mg/kg. The inhibitory effect caused by ortho and meta at the 100 mg/kg dosage was significant 10 min after administration of the compounds. Para and tramadol doses of 100 mg/kg were effective 60 min after administration. Systemic administration of PCP (0.01–10 mg/kg) dose-dependently inhibited acoustic responses in mice. PCP data are replicated from [17].

A comparison of the maximal effect among 1-cyclohexyl-x-methoxybenzene derivatives, tramadol and PCP (Figure 5E) revealed significant differences between the effects of these compounds ((significant effect of treatment (F_4,175_ = 40.14; *p* < 0.0001), dose (F_4,175_ = 137.4; *p* < 0.0001) and dose x treatment interaction (F_16,175_ = 4.959; *p* < 0.0001)). Notably, ortho, meta and tramadol doses of 1 mg/kg were more effective in inhibiting acoustic responses (*p* < 0.05) in mice. Pretreatment with naloxone 6 mg/kg did not prevent the inhibitory effect induced by ortho, meta, para, tramadol (100 mg/kg) and 10 mg/kg of PCP (Figure 5F).

##### Evaluation of the Core Body Temperature

**Figure 6:**
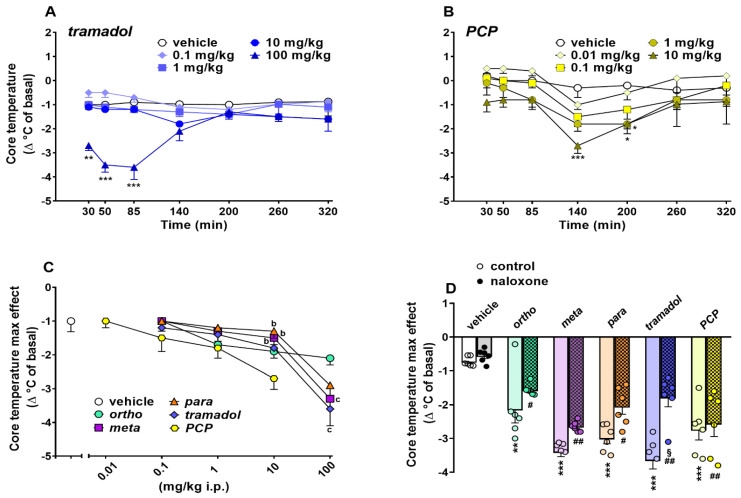
Effects of the systemic administration of tramadol (0.1–100 mg/kg i.p.; (**A**)) and PCP (0.01–10 mg/kg i.p.; (**B**)) on the mouse core temperature. Comparison of the maximal effect of ortho, meta, para, tramadol (0.1–100 mg/kg) and PCP (0.01–10 mg/kg) observed in 5 h (**C**) ^1^. Interaction of the effects of ortho, meta, para, tramadol (100 mg/kg) and PCP (10 mg/kg) with the opioid receptor antagonist naloxone (6 mg/kg, i.p.; (**D**)). Data are expressed as the difference between control temperature (before injection) and temperature following drug administration (Δ°C; see material and methods), and represent the mean ± SEM of 6–8 determinations for each treatment. Statistical analysis was performed by two-way ANOVA followed by the Bonferroni’s test for multiple comparisons for the dose–response curve of each compound at different times (**A**,**B**), and for the antagonist studies (**D**), while the statistical analysis of (**C**) was performed with one-way ANOVA followed by Bonferroni test for multiple comparisons. * *p* < 0.05, ** *p* < 0.01, *** *p* < 0.001 versus vehicle; ^b^ *p* < 0.05 versus PCP; ^c^ *p* < 0.05 versus ortho; ^#^ *p* < 0.05, ^##^ *p* < 0.01 versus naloxone; ^§^ *p* < 0.05 versus without naloxone. ^1^ ortho, meta and para data are elaborated from [5].

Core body temperature did not change in vehicle-treated mice over 5 h of observation (Figure 6A,B) and the effect was similar to that observed in naïve untreated animals (data not shown). Systemic administration of 1-cyclohexyl-x-methoxybenzene derivatives (0.1–100 mg/kg) dose-dependently affected core temperature in mice. Data of 1-cyclohexyl-x-methoxybenzene derivatives (ortho, meta and para) are replicated from [5].

Systemic administration of tramadol (0.1–100 mg/kg, i.p.; Figure 6A; significant effect of treatment (F_4,245_ = 26.31; *p* < 0.0001), time x treatment interaction (F_28,245_ = 2.588; *p* = 0.0001) but not time (F_7,245_ = 1.044; *p* = 0.3976)) and PCP (0.01–10 mg/kg, i.p.; Figure 6B; significant effect of treatment (F_4,245_ = 10.77; *p* < 0.0001), time (F_7,245_ = 7.55: *p* < 0.0001) but not time x treatment interaction (F_28,245_ = 0.4589; *p* = 0.9871)) transiently reduced the core body temperature in mice. Hypothermia caused by tramadol at 100 mg/kg was evident after 30 min and reached maximum effect at 85 min (Δ°C = ~−3.6 °C; Figure 6A), while the hypothermic effect caused by PCP at 10 mg/kg was significant and reached a maximum after 140 min (Δ°C = ~−2.7 °C; Figure 6B). A comparison of the maximal effect among 1-cyclohexyl-x-methoxybenzene derivatives, tramadol and PCP (Figure 6C) revealed significant differences ((significant effect of dose (F_4,175_ = 47.96; *p* < 0.0001) but not treatment (F_4,175_ = 1.491; *p* = 0.2070) and dose x treatment interaction (F_16,175_ = 1.415; *p* = 0.1393)). Notably, PCP transiently induced hypothermia in mice at both 1 and 10 mg/kg (*p* < 0.05). Moreover, the meta and tramadol at 100 mg/kg were more effective with respect to ortho regarding the induction of hypothermia in mice (*p* < 0.05). Pretreatment with naloxone 6 mg/kg did not prevent the reduction in body core temperature induced by ortho, meta, para (100 mg/kg) and PCP 10 mg/kg, while it partially prevented that caused by tramadol 100 mg/kg (Figure 6D).

##### Evaluation of Pain Induced by Mechanical and Thermal Stimuli

**Figure 7:**
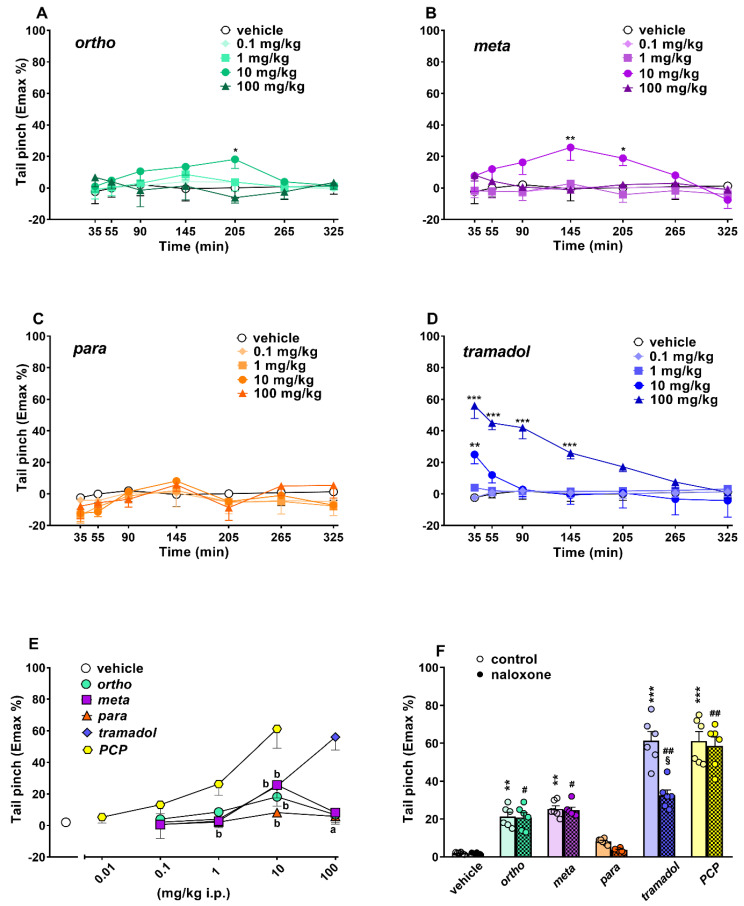
Effects of the systemic administration (0.1-100 mg/kg i.p.) of ortho (**A**), meta (**B**), para (**C**) and tramadol (**D**) on the tail pinch test in mice. Comparison of the maximal effect of ortho, meta, para, tramadol (0.1–100 mg/kg) and PCP (0.01–10 mg/kg) observed in 5 h (**E**) ^2^. Interaction of the effects of ortho, meta, para, tramadol (100 mg/kg) and PCP (10 mg/kg) with the opioid receptor antagonist naloxone (6 mg/kg, i.p.; (**F**)). Data are expressed as percentage of maximum effect (Emax%; see material and methods) and represent the mean ± SEM of 6–8 determinations for each treatment. Statistical analysis was performed by two-way ANOVA, followed by the Bonferroni’s test for multiple comparisons for the dose–response curve of each compound at different times (**A**–**D**), and for the antagonist studies (**E**), while the statistical analysis of panel F was performed with one-way ANOVA followed by Bonferroni test for multiple comparisons. * *p* < 0.05, ** *p* < 0.01, *** *p* < 0.001 versus vehicle; ^a^ *p* < 0.05 versus tramadol; ^b^ *p* < 0.05 versus PCP; ^#^ *p* < 0.05, ^##^ *p* < 0.01 versus naloxone; ^§^ *p* < 0.05 versus without naloxone; ^2^ PCP data are elaborated from [17].

The threshold of mechanical pain did not change in vehicle-treated mice over 5 h of observation (Figure 7A–D), and the effect was similar to that observed in naïve untreated animals (data not shown). Systemic administration (0.1–100 mg/kg i.p.) of ortho (Figure 7A; significant effect of treatment (F_4,245_ = 2.467, *p* = 0.0455) but not time (F_6,245_ = 0.6174, *p* = 0.7163) and time x treatment interaction (F_24,245_= 0.6098, *p* =0.9252)), meta (Figure 7B; significant effect of treatment (F_4,245_ =7.664, *p* < 0.0001) but not time (F_6,245_ = 1.073, *p* = 0.3792) and time x treatment interaction (F_24,245_ = 0.9550, *p* = 0.5270)) and tramadol (Figure 7D; significant effect of treatment (F_4,245_ = 39.08, *p* < 0.0001), time (F_6,245_ = 6.759, *p* < 0.0001) and time x treatment interaction (F_24,245_ = 3.762, *p* < 0.0001)) increased the threshold of mechanical pain in the pinch test in mice. However, the para compound was ineffective (Figure 7C).

Ortho and meta at 10 mg/kg transiently and mildly induced mechanical analgesia after 205 (ortho Emax ~18%) and 145 (meta Emax ~25%) minutes, respectively, while at the highest dose (100 mg/kg), both compounds were ineffective. However, tramadol induced a rapid increase in mechanical analgesia at 10 mg/kg (Emax ~25%) and 100 mg/kg (Emax ~56%; Figure 7D), and the effect of tramadol 100 mg/kg persisted for up to 145 min. Systemic administration of PCP (0.01–10 mg/kg) dose-dependently induced mechanical analgesia in mice. PCP data are replicated from [17].

A comparison of the maximal effect among 1-cyclohexyl-x-methoxybenzene derivatives, tramadol and PCP (Figure 7E) revealed significant differences between the effects of these compounds ((significant effect of treatment (F_4,175_ = 10.98; *p* < 0.0001), dose (F_4,175_ = 21.74; *p* < 0.0001) and dose x treatment interaction (F_16,175_ = 5.517; *p* < 0.0001)). PCP was the most potent compound, while tramadol 100 mg/kg was more effective with respect to ortho and meta derivatives. Pretreatment with naloxone 6 mg/kg did not prevent the analgesic effect induced by ortho, meta and PCP (10 mg/kg), while it partially prevented that caused by tramadol 100 mg/kg (Figure 7F).

**Figure 8:**
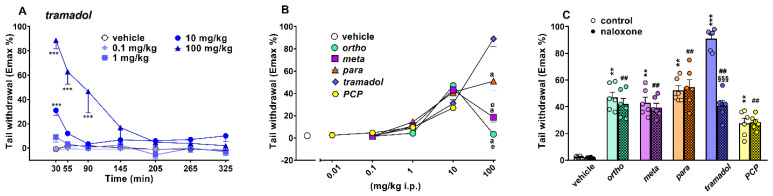
Effect of the systemic administration of tramadol (0.1–100 mg/kg i.p.; (**A**)) on the tail withdrawal test in mice. Comparison of the maximal effect of ortho, meta, para, tramadol (0.1–100 mg/kg) and PCP (0.01–10 mg/kg) observed over 5 h (**B**) ^1,2^. Interaction of the maximal effective dose of ortho, meta, para, tramadol (100 mg/kg) and PCP (10 mg/kg) with the opioid receptor antagonist naloxone (6 mg/kg, i.p.; (**C**)). Data are expressed as percentage of maximum effect (Emax%; see material and methods) and represent the mean ± SEM of 6–8 determinations for each treatment. Statistical analysis was performed by two-way ANOVA, followed by Bonferroni’s test for multiple comparisons for the dose–response curve of each compound at different times (**A**), and for the antagonist studies (**C**), while the statistical analysis of (**B**) was performed with one-way ANOVA followed by Bonferroni test for multiple comparisons. ** *p* < 0.01, *** *p* < 0.001 versus vehicle; ^a^ *p* < 0.05 versus tramadol; ^e^ *p* < 0.05 versus para; ^##^ *p* < 0.01 versus naloxone; ^§§§^ *p* < 0.001 versus without naloxone; ^1^ ortho, meta and para data are elaborated from [5]; ^2^ PCP data are elaborated from [17].

The threshold of thermal pain did not change in vehicle-treated mice over 5 h of observation (Figure 8A) and the effect was similar to that observed in naïve untreated animals (data not shown). Systemic administration of 1-cyclohexyl-x-methoxybenzene derivatives (0.1–100 mg/kg) and PCP (0.1–10 mg/kg) dose-dependently affected the threshold of thermal pain in mice. Data of 1-cyclohexyl-x-methoxybenzene derivatives (ortho, meta and para) are replicated from [5], and PCP data are replicated from [17].

Systemic administration of tramadol (0.1–100 mg/kg i.p.) increased the threshold for thermal pain in the tail withdrawal test in mice (Figure 8A; significant effect of treatment (F_4,245_ = 57.54, *p* < 0.0001), time (F_6,245_ = 17.84, *p* < 0.0001) and time x treatment interaction (F_24,245_ = 8.908, *p* < 0.0001)). Tramadol induced a rapid increase in the thermal analgesia at 10 mg/kg (Emax ~31%) and 100 mg/kg (Emax ~89%; Figure 8A), and the effect of tramadol 100 mg/kg persisted for up to 90 min.

A comparison of the maximal effect among 1-cyclohexyl-x-methoxybenzene derivatives, tramadol and PCP (Figure 8B) revealed significant differences between the effects of these compounds ((significant effect of treatment (F_4,175_ =11.36; *p* < 0.0001), dose (F_4,175_ = 122.7; *p* < 0.0001) and dose x treatment interaction (F_16,175_ = 17.27; *p* < 0.0001)). Tramadol was the more effective compound with respect to the 1-cyclohexyl-x-methoxybenzene derivatives. Pretreatment with naloxone 6 mg/kg did not prevent the analgesic effect induced by ortho, meta, para and PCP (10 mg/kg), while it partially prevented that caused by tramadol 100 mg/kg (Figure 8C).

##### Bar Test

The time spent on the bar did not change in vehicle-treated mice over 5 h of observation (data not shown), and the effect was similar to that observed in naïve untreated animals (data not shown). The systemic administration of ortho, meta, para, tramadol (0.1–100 mg/kg i.p.) and PCP (0.01–10 mg/kg i.p.) did not induce akinesia and did not affect the time spent on bar (data not shown).

##### Accelerod Test

**Figure 9:**
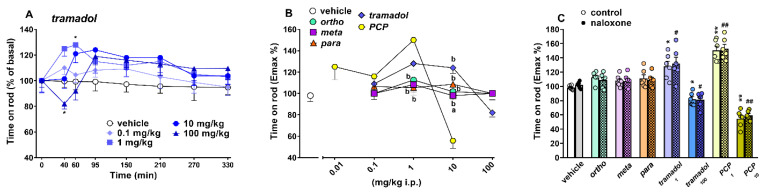
Effect of the systemic administration of tramadol (0.1–100 mg/kg i.p.; (**A**) on the accelerod test in mice. Comparison of the maximal effect of ortho, meta, para, tramadol (0.1–100 mg/kg) and PCP (0.01–10 mg/kg) observed in 5 h (**B**) ^1,2^. Interaction of the maximal effective doses of ortho, meta, para (100 mg/kg), tramadol (1 and 100 mg/kg) and PCP (1 and 10 mg/kg) with the opioid receptor antagonist naloxone (6 mg/kg, i.p.; (**C**)). Data are expressed as percentage of basal and represent the mean ± SEM of 6–8 determinations for each treatment. Statistical analysis was performed by two-way ANOVA, followed by the Bonferroni test for multiple comparisons for the dose–response curve of each compound at different times (**A**), and for the antagonist studies (**C**), while the statistical analysis of (**B**) was performed with one-way ANOVA followed by Bonferroni test for multiple comparisons. * *p* < 0.05, ** *p* < 0.01, versus vehicle; ^a^ *p* < 0.05 versus tramadol; ^b^ *p* < 0.05 versus PCP; ^#^ *p* < 0.05, ^##^ *p* <0.01 versus naloxone; ^1^ ortho, meta and para data are elaborated from [5]; ^2^ PCP data are elaborated from [17].

The time spent on the accelerod did not change in vehicle-treated mice over 5 h of observation (Figure 9A,) and the effect was similar to that observed in naïve untreated animals (data not shown). Systemic administration of 1-cyclohexyl-x-methoxybenzene derivatives (0.1–100 mg/kg; [5]) and PCP (0.1–10 mg/kg; [17]) dose-dependently affected motor activity on the accelerod in mice. Data of 1-cyclohexyl-x-methoxybenzene derivatives (ortho, meta and para) are replicated from [5], and PCP data are replicated from [17].

Systemic administration of tramadol transiently modulated the motor activity in the accelerod test in mice ((significant effect of treatment (F_4,280_ = 5.502; *p* = 0.0003), time (F_7,280_ = 2.125; *p* = 0.0411) but not time x treatment interaction (F_28,280_ = 1.257; *p* = 0.1802)). In particular, tramadol transiently facilitated the stimulated motor activity of mice on the accelerod at 1 mg/kg (~28% of basal activity at 60 min timepoint) and inhibited it at 100 mg/kg (~20% of basal activity at 40 min timepoint) (Figure 9A).

A comparison of the maximal effect among 1-cyclohexyl-x-methoxybenzene derivatives, tramadol and PCP (Figure 9B) revealed significant differences between the motor effects of these compounds ((significant effect of treatment (F_4,175_ = 36.18; *p* = 0.0077), dose (F_4,175_ = 25.56; *p* < 0.0001) and dose x treatment interaction (F_16,175_ = 10.85; *p* < 0.0001)). Notably, PCP was more effective than tramadol in facilitating (at 1 mg/kg) and inhibiting (10 mg/kg) the motor coordination of mice on the accelerod test. The 1-cyclohexyl-x-methoxybenzene derivatives were inactive in this motor test [5]). Pretreatment with naloxone 6 mg/kg did not prevent the facilitatory and inhibitory motor effects caused by both tramadol (1 and 100 mg/kg, respectively) and PCP 1 and 10 mg/kg, respectively (Figure 9C).

##### Spontaneous Locomotion Test

**Figure 10:**
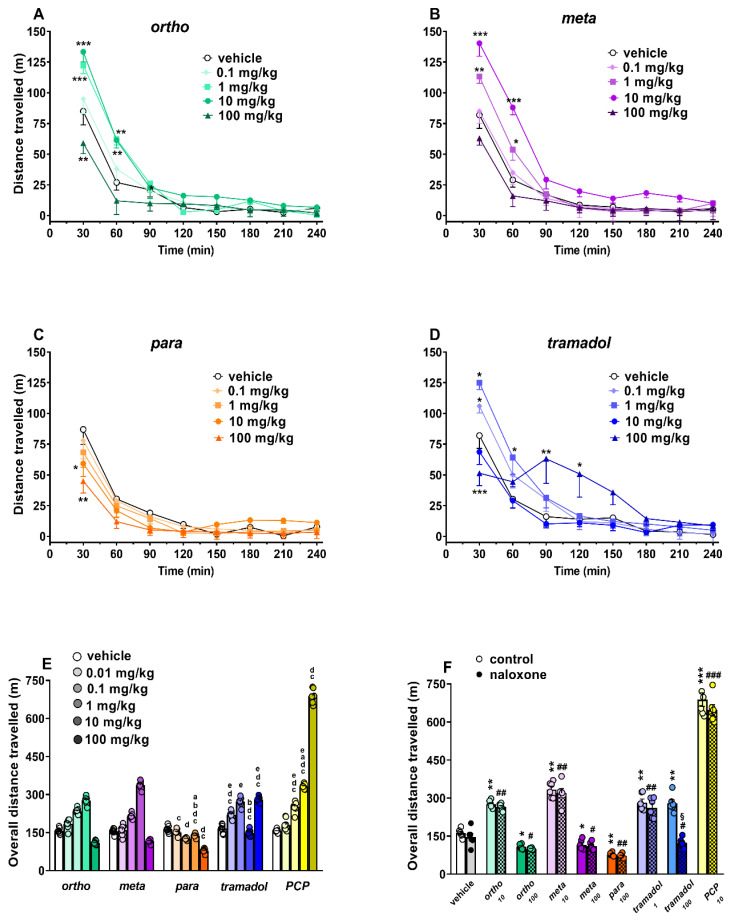
Effects of the systemic administration (0.1–100 mg/kg i.p.) of ortho (**A**), meta (**B**), para (**C**) and tramadol (**D**) on the total distance travelled of the mouse. Representation of the effect induced by ortho, meta, para, tramadol (0.1–100 mg/kg) and PCP (0.01–10 mg/kg) treatments on the overall distance traveled observed in 4 h (**E**) ^2^. Interaction of the effective doses of ortho (10 and 100 mg/kg), meta (10 and 100 mg/kg), para (100 mg/kg), tramadol (1 and 100 mg/kg) and PCP (10 mg/kg) with the opioid receptor antagonist naloxone (6 mg/kg, i.p.; (**F**)). Data are expressed as meters travelled (total distance travelled) and represent the mean ± SEM of 8 determinations for each treatment. Statistical analysis was performed by two-way ANOVA, followed by Bonferroni’s test for multiple comparisons of the dose–response curve of each compound at different times (**A**–**D**), and for the antagonist studies (**F**), while the statistical analysis of panel E was performed with one-way ANOVA followed by Bonferroni test for multiple comparisons. * *p* < 0.05, ** *p* < 0.01, *** *p* < 0.001 versus vehicle; ^a^ *p* < 0.05 versus tramadol; ^b^ *p* < 0.05 versus PCP; ^c^ *p* < 0.05 versus ortho; ^d^ *p* < 0.05 versus meta; ^e^ *p* < 0.05 versus para; ^#^ *p* < 0.05, ^##^ *p* < 0.01, ^###^ *p* < 0.001 versus naloxone; ^§^ *p* < 0.05 versus without naloxone. ^2^ PCP data are elaborated from [17].

Spontaneous locomotion was affected by the systemic administration of ortho (Figure 10A; significant effect of treatment (F_4,280_ = 15.26, *p* < 0.0001), time (F_7,280_ = 152.7, *p* < 0.0001) and time x treatment interaction (F_28,280_ = 3.886, *p* < 0.0001)), meta (Figure 10B; significant effect of treatment (F_4,280_ = 22.99, *p* < 0.0001), time (F_7,280_ = 131.6, *p* < 0.0001) and time x treatment interaction (F_28,280_ = 3.599, *p* < 0.0001)) para (Figure 10C; significant effect of treatment (F_4,280_ = 3.22, *p* = 0.0132), time (F_7,280_ = 55.98, *p* < 0.0001) and time x treatment interaction (F_28,280_ = 1.003, *p* = 0.4651)) and tramadol (Figure 10C; significant effect of treatment (F_4,280_ = 6.908, *p* < 0.0001), time (F_7,280_ = 58.63, *p* < 0.0001) and time x treatment interaction (F_28,280_ = 3.377, *p* < 0.0001)). In particular, 1-cyclohexyl-x-methoxybenzene derivatives differently affected spontaneous motor activity. Ortho (Figure 10A) transiently facilitated spontaneous locomotion in mice at 1 (~+43% at 30 min) and 10 (~+56%) mg/kg, while, at the highest dose (100 mg/kg), it inhibited (~−30%) mouse motor activity. Meta (Figure 10B) transiently facilitated spontaneous locomotion in mice at 1 (~+38% at 30 min) and 10 (~+70%) mg/kg, while para (Figure 10C) inhibited it at 10 (~−32% at 30 min) and 100 (~−48%) mg/kg. Tramadol (Figure 10D) facilitated spontaneous locomotion in mice at lower doses of 0.1 (~+29% at 30 min) and 1 (~+52%) mg/kg, while at the highest dose (100 mg/kg), it inhibited (~−38%) mouse motor activity. As previously reported, the systemic administration of PCP (0.01–10 mg/kg) facilitated spontaneous locomotion in mice. PCP data are replicated from [17].

A comparison of the overall distance travelled among 1-cyclohexyl-x-methoxybenzene derivatives, tramadol and PCP, analyzed at the same dose range (Figure 10E), revealed significant differences among the motor effects of these compounds. In particular, tramadol and PCP were more potent in facilitating spontaneous locomotion with respect to 1-cyclohexyl-x-methoxybenzene derivatives (F_4,45_ = 32.06; *p* < 0.0001), since they were effective at 0.1 mg/kg. Moreover, statistical analysis revealed that PCP is the most effective compound in stimulating locomotion in mice, at both 1 mg/kg (F_4,45_ =58.18; *p* < 0.0001) and 10 mg/kg (F_4,45_ = 217; *p* < 0.0001). However, para was the more effective compound in inhibiting spontaneous locomotion at the highest dose tested (100 mg/kg; F_3,36_ = 596; *p* < 0.0001).

Pretreatment with naloxone 6 mg/kg did not prevent the motor effects caused by both 1-cyclohexyl-x-methoxybenzene derivatives and PCP (Figure 10F). It is interesting to note that, while the facilitating effect of tramadol at 1 mg/kg is insensitive to naloxone, the facilitating effect induced by tramadol at 100 mg/kg in the time window ranging from 90 to 120 min is prevented by treatment with naloxone.

##### Cardiorespiratory Analysis

**Figure 11:**
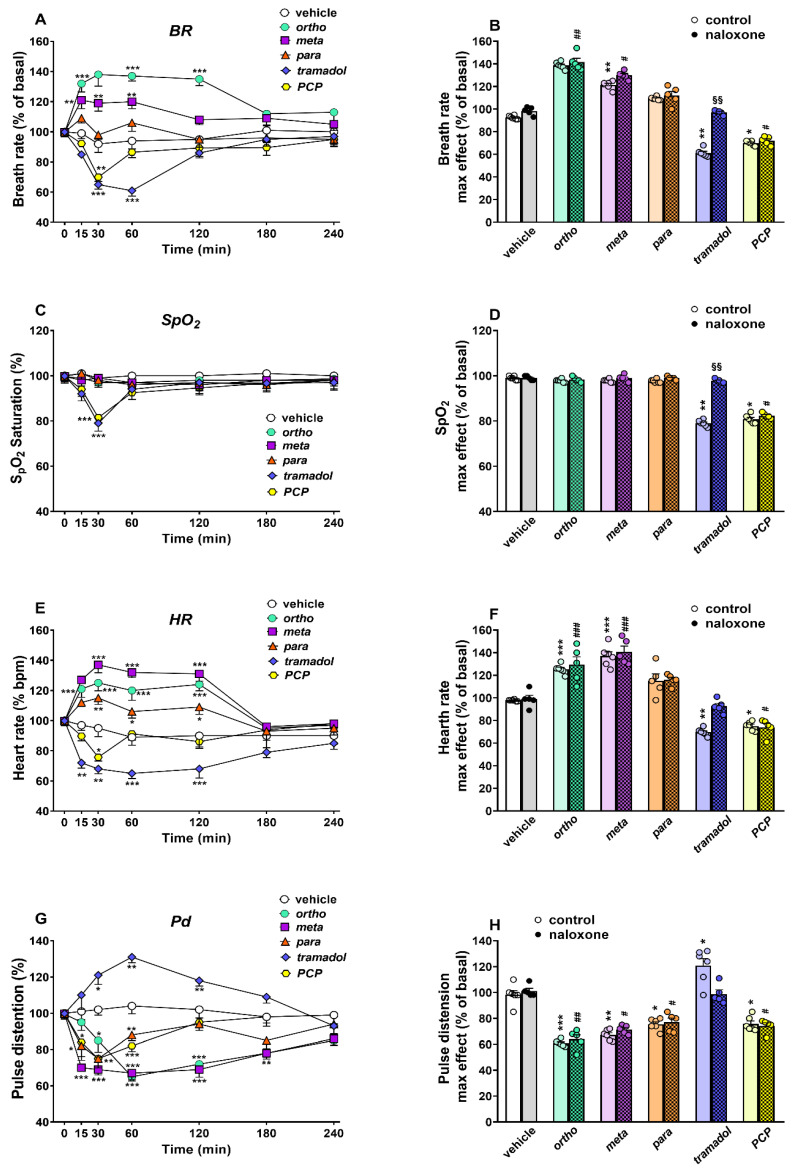
Effect of the systemic administration of ortho, meta, para, tramadol (100 mg/kg i.p.) and PCP (10 mg/kg i.p.) on breath rate (BR; (**A**)), on arterial saturation (SpO2; (**C**)), heart rate (HR; (**E**)) and pulse distention (Pd; (**G**)). Interaction of the effective doses of ortho, meta, para, tramadol (100 mg/kg) and PCP (10 mg/kg) with the opioid receptor antagonist naloxone (6 mg/kg, i.p.) on breath rate (BR; (**B**)), arterial saturation (SpO2; (**D**)), heart rate (HR (**F**)) and pulse distention (Pd; (**H**)). Data are expressed as percentage of basal value (BR, HR and Pd), while absolute values for oxygen blood saturation (% SpO2 saturation) are represent as the mean ± SEM of 6–5 determinations for each treatment. Statistical analysis was performed by two-way ANOVA followed by Bonferroni’s test for multiple comparisons for both the dose–response curve of each compound at different times ((**A**,**C**,**E**,**G**)) and interactions with naloxone (**B**,**D**,**F**,**H**). * *p* < 0.05, ** *p* < 0.01, *** *p* < 0.001 versus vehicle; ^#^ *p* < 0.05, ^##^ *p* < 0.01 ^###^ *p* < 0.001 versus naloxone; ^§§^ *p* < 0.01 versus without naloxone.

To investigate if treatment with highest doses of the 1-cyclohexyl-x-methoxybenzene derivatives (100 mg/kg), tramadol (100 mg/kg) and PCP (10 mg/kg) can modify the normal cardiorespiratory pattern of mice, we used the MouseOX instrument (see Materials and Methods). Vehicle administration did not affect the basal breath rate (BR, 175 ± 15 brpm; Figure 11A), oxygen saturation (SpO2, 99.5 ± 1.2%; Figure 11C), heart rate (HR, 570 ± 15 bpm; Figure 11E) and pulse distention (Pd, 243 ± 17 µm; Figure 11G) of mice during the 4 h of measuring. Administration of the 1-cyclohexyl-x-methoxybenzene derivatives (100 mg/kg), tramadol (100 mg/kg) and PCP (10 mg/kg) affected the basal BR in mice (Figure 11A; significant effect of treatment (F_5,210_ = 69.21, *p* < 0.0001), time (F_6,210_ = 2.15, *p* = 0.0491) and time x treatment interaction (F_30,210_ = 5.773, *p* < 0.0001)). Notably, BR was transiently increased by ortho (max effect ~ +38% of basal values at 30 min) and meta (max effect ~ +21% of basal values at 15 min), but not para, administration, while BR was transiently reduced by tramadol (max effect ~−40% of basal values at 60 min) and PCP (max effect ~−30% of basal values at 30 min). Pretreatment with naloxone 6 mg/kg completely prevented the bradypnea induced by tramadol, but was ineffective in blocking the effects caused by ortho, meta and PCP administration (Figure 11B).

The administration of tramadol (100 mg/kg) and PCP (10 mg/kg), but not 1-cyclohexyl-x-methoxybenzene derivatives (100 mg/kg), affected the basal SpO2 in mice (Figure 11C; significant effect of treatment (F_5,210_ = 6.12, *p* < 0.0001), time (F_6,210_ = 4.281, *p* = 0.0004) and time x treatment interaction (F_30,210_ = 1.452, *p* = 0.0695)). Notably, SpO2 was transiently reduced by tramadol (max effect ~−20% of SpO2 saturation at 30 min) and PCP (max effect ~−19% of SpO2 saturation at 30 min), and their inhibitory effects disappeared 60 min after compound administration. Pretreatment with naloxone 6 mg/kg completely prevented the reduction in SpO2 induced by tramadol, but was ineffective in blocking the effects of PCP administration (Figure 10D).

The administration of 1-cyclohexyl-x-methoxybenzene derivatives (100 mg/kg), tramadol (100 mg/kg) and PCP (10 mg/kg) affected the basal HR in mice (Figure 11E; significant effect of treatment (F_5,210_ = 82.51, *p* < 0.0001), time (F_6,210_ = 6.673, *p* < 0.0001) and time x treatment interaction (F_30,210_ = 7.413, *p* < 0.0001)). In particular, HR was transiently but maximally increased at 30 min by ortho (max effect ~+25% of basal values), meta (max effect ~+37% of basal values) and para (max effect ~+15% of basal values), and the effects persisted for up to 120 min. Conversely, HR was mildly and transiently reduced by PCP (max effect ~−25% of basal values at 30 min), and was reduced for up to 120 min by tramadol (max effect ~−35% of basal values at 60 min). Pretreatment with naloxone 6 mg/kg did not prevent the increase in HR in mice that was induced by 1-cyclohexyl-x-methoxybenzene derivatives, or the inhibition caused by PCP administration, while naloxone prevented the inhibition induced by tramadol (Figure 11F).

The administration of the 1-cyclohexyl-x-methoxybenzene derivatives (100 mg/kg), tramadol (100 mg/kg) and PCP (10 mg/kg) affected the basal Pd in mice (Figure 11G; significant effect of treatment (F_5,210_ = 61.59, *p* < 0.0001), time (F_6,210_ = 4.881, *p* = 0.0001) and time x treatment interaction (F_30,210_ = 6.055, *p* < 0.0001)). Notably, Pd was maximally reduced at 30 min by ortho (max effect ~ −35% of basal values at 60 min), meta (max effect ~−33% of basal values at 60 min), para (max effect ~−25% of basal values at 30 min), and PCP (max effect ~−25% of basal values at 30 min). The reduction in the Pd persisted for the ortho and meta for up to 180 min and for para and PCP up to 60 min. Conversely, tramadol increased the Pd in mice (max effect ~ +31% of basal values at 60 min), and its effect lasted for up to 120 min. Pretreatment with naloxone 6 mg/kg did not prevent the reduction in Pd in mice induced by 1-cyclohexyl-x-methoxybenzene derivatives and PCP administration, while it prevented the increase induced by tramadol (Figure 11H).

## 3. Discussion

Our study presents novel results regarding the in vitro and in vivo characterization of the 1-cyclohexyl-x-methoxybenzene stereoisomers. Using the calcium mobilization assay, we demonstrated in vitro that the three stereoisomers, similar to tramadol, were inactive at mu, kappa and delta human recombinant receptors coupled with calcium signaling via chimeric G proteins. Conversely, the in vivo study provides the first direct comparison of the effects of 1-cyclohexyl-x-methoxybenzene stereoisomers and those of the two most similar compounds, tramadol and PCP [3], and demonstrates that ortho, meta and para stereoisomers impair acoustic and visual sensorimotor responses, induce analgesia, modulate motor activity and affect cardiorespiratory functions in mice. The three stereoisomers show a similar profile of action, with slight differences in potency and efficacy, probably due to their different substitution on the benzyl ring of the methoxylic group [5]. The effects caused by 1-cyclohexyl-x-methoxybenzene derivatives are generally qualitatively similar to those induced by tramadol and/or PCP administration, and in some tests, partially blocked by naloxone administration (see Table 2), suggesting the involvement of the opioid receptor system.

### 3.1. Major Neurological Changes

The administration of up to 100 mg/kg of three stereoisomers does not induce major neurological alterations, such as tail elevation, hyperreflexia and convulsions, in mice, highlighting their lower toxicity than tramadol and PCP. In fact, tramadol at 100 mg/kg causes tail elevation and convulsions in 90% of mice, while PCP at 100 mg/kg causes the rapid death of all treated mice.

Convulsions are typically reported in tramadol overdose in both animals and humans and, together with respiratory depression, they represent the most serious and dangerous aspect of acute tramadol poisoning [18,19,20,21,22,23]. Our data are consistent with previous reports showing that naloxone does not antagonize tail elevation or even augment tramadol-induced seizures in rodents [22,24]. This aspect is very important, as it highlights that incorrect antidotal therapy can worsen the symptoms of intoxication. However, the administration of naloxone is ineffective or only partially effective in preventing the effects of the 1-cyclohexyl-x-methoxybenzene derivatives and, fortunately, it does not worsen their pharmaco-toxicological effects.

### 3.2. Effect on Sensorimotor Responses

The present study confirms and extends previous evidence showing that 1-cyclohexyl-x-methoxybenzene derivatives impair visual sensorimotor responses in mice [5]. Notably, these compounds impair visual perception under both “static” (visual object response; [5]) and “dynamic” (visual placing response, present data) conditions, and reduce similar acoustic responses to tramadol and PCP [17]. Notably, the meta compound at 1 mg/kg appears to be more effective than PCP and tramadol in reducing visual and acoustic sensorimotor responses, suggesting it may have potential detrimental effect on driving and other human activities that require skill and attention.

The pharmacological response induced by the stereoisomers on sensorimotor responses is more similar to that caused by tramadol than that induced by PCP, having a prolonged effect, characterized by a slow appearance, with a maximum effect at 120 min which lasts up to 5 h. Otherwise, PCP causes a transient, rapid and maximum impairment of the visual and acoustic sensorimotor responses in the first 10–30 min, which reverts quickly and is extinguished in 130–180 min (visual impairments) or 60 min (acoustic alterations) for doses lower than 10 mg/kg [17].

The sensorimotor alteration induced by tramadol (as racemic mixture) could be due to its different opioid and non-opioid pharmacodynamic activity. The opioid component, which is predominant in the (+) enantiomer, has a weak affinity for µ opioid receptors, while its metabolite [(+)-Odesmethyl-tramadol] was about 300-fold more potent at mu opioid receptors [25]. The non-opioid component, predominant in the (−) enantiomer, is related to the inhibition of neuronal serotonin (5-HT) and norepinephrine (NE) reuptake [22], and also to the non-competitive antagonism of NMDA and GABAA receptors [26].

Therefore, these mechanisms could be involved in the visual sensorimotor alterations of tramadol; particularly the opioid component, since visual sensorimotor alterations are partially prevented by blocking opioid receptors with naloxone. In vitro results suggest a very low potency of tramadol at opioid receptors. However, it should be noted that tramadol is metabolized to O-Desmethyltramadol (O-Dt), a more potent mu agonist [25]. In fact, a differential study of the brain uptake of tramadol and its metabolite O-Dt showed that the peak brain levels of the two compounds coincide at higher dosage (40 mg/kg; [27]). Thus, the O-Dt metabolite of tramadol could be responsible for the neurological (particularly tail elevation) and sensorimotor alterations that are, at least in part, sensitive to naloxone.

Accordingly, data from our laboratory showed that stimulation of the opioid receptor by morphine or fentanyl impairs visual object and visual placing responses in mice in a dose-dependent and naloxone-sensitive manner, and this effect is possibly due to the activation of opioid receptors in brain areas controlling both visual and vestibular function [28,29].

An increase in serotonergic and noradrenergic transmission may also contribute to alterations in the visual sensorimotor. In fact, tramadol, by increasing 5-HT levels, may induce visual alterations via the activation of serotonin receptors in the cortico-visual circuits [30,31]. In particular, the inhibition of both visual object and visual placing responses in rodents has been observed after the systemic administration of MDMA [32], DOB [33] or the potent serotoninergic agonist 25I-NBOMe [34].

The increase in the noradrenergic signal could contribute to altering the sensorimotor responses in the visual placing test, and not the visual object test. This differing response is due to the fact that NE does not alter the perception of visual stimuli (i.e., visual object response), but possibly causes an alteration in the vestibular signals involved in the correct execution of the visual placing test (see [35]). Therefore, tramadol, by releasing NE, could change the vestibulo-ocular reflex and the optokinetic response in mice through β- and α2-receptor activation [36], thus impairing visual placing responses.

The antagonism on NMDA receptors exerted by tramadol [26] should also be considered. NMDA-receptor blockade caused inhibitions in visual sensory motor responses in mice, as reported for PCP and other dissociative drugs (i.e., ketamine, methoxetamine [17]).

Therefore, 1-cyclohexyl-x-methoxybenzene derivatives could impair visual object and visual placing responses by recruiting both opioid (naloxone-sensitive) and non-opioid mechanisms such as tramadol and PCP, and be used to alter visual perception.

The three stereoisomers (particularly ortho and meta) induce a significant and long-lasting impairment of the acoustic startle responses in mice, similarly to tramadol (100 mg/kg), but not PCP, which causes a rapid but transient inhibition of acoustic responses (10 mg/kg; [17]). The acoustic impairment was naloxone-resistant for all the tested compounds. Recently, the role of serotonin in modulating auditory brainstem responses has been demonstrated in mice, starting from the cochlear nucleus [37]. Indeed, in the dorsal region of this nucleus, the activation of 5HT2 receptors acts by increasing the electrical activity of neurons, leading to the final suppression of auditory process [38,39]. The exclusion of the possible involvement of opioid receptors in acoustic responses is supported by the fact that, in addition to the naloxone-resistant action reported in this study, morphine or the synthetic opioid MT-45 are ineffective in altering auditory sensorimotor responses, although they reduce visual responses in CD-1 mice [28]. Therefore, the fact that the three stereoisomers alter sensorimotor responses must be well-studied.

### 3.3. Effect on Spontaneous and Stimulated Motor Activity

The ortho and meta stereoisomers facilitate spontaneous locomotion in mice at low doses (0.1 and 1 mg/kg), and inhibit it at higher doses (100 mg/kg) spontaneous, while the para stereoisomer only inhibits motor activity. These motor effects are independent of opioid receptor stimulation, as they are not prevented by naloxone administration. The facilitation of spontaneous (open field) but not “stimulated” locomotion (rotarod test; [5]) suggests that these stereoisomers probably do not promote DA release in motor brain areas, even if administered at a higher dose (100 mg/kg). In fact, as reported for some typical DA/NEergic psychostimulants, such as methylone, butylone [40], α-PVP, MDPV [41] and methiopropamine [35,36,37,38,39,40,41,42], the robust stimulation of spontaneous motor activity is associated with an improvement in the rotarod test performance, and with the appearance of stereotypies and turning behavior at the highest tested dose, which impairs motor performance in mice [35,41]. It is interesting to note that the inhibition of spontaneous locomotion caused by the highest dose of 1-cyclohexyl-x-methoxybenzene derivatives (100 mg/kg) is not due to akinesia (no effect in the bar test), or the appearance of stereotypies and turning behavior. Therefore, this motor inhibition could possibly be due to either an anxiogenic effect of the molecule (stimulation of the noradrenergic fight-or-flight response) or as a consequence of the impaired visual and acoustic sensorimotor responses. In fact, the systemic administration of hallucinogenic phenethylamines that act as 5-HT receptor agonists, such as DOB, 2C-I and 25I-NBOMe [43], profoundly inhibits visual and acoustic sensorimotor responses in CD-1 mice, inducing a mild inhibition of spontaneous locomotion [33].

Differently from 1-cyclohexyl-x-methoxybenzene derivatives, tramadol and PCP [17] affect both spontaneous and stimulated motor activity in mice. Notably, low doses (0.1 and 1 mg/kg) of tramadol facilitate spontaneous locomotion and increase mouse performance on the accelerod (1 mg/kg). At the highest dose (100 mg/kg), tramadol biphasically modulated spontaneous locomotion by producing a transient (30 min) inhibition, followed by a long-lasting (>2 h) stimulation.

Conversely, on the accelerod test, tramadol 100 mg/kg transiently inhibited mice motor performance in the first 40 min, as observed for spontaneous locomotion. The facilitation of locomotion at low doses is independent of the stimulation of opioid receptors, while the modulation of motor activity at 100 mg/kg is partially prevented by naloxone. In particular, naloxone prevented the facilitation of spontaneous locomotion observed in the second phase (from 90 min to 130 min), but did not prevent the inhibitory effect observed in the first 30 min. This naloxone-insensitive impaired locomotion is observed on both spontaneous and stimulated locomotion on the accelerod (in line with that reported by [24]) and could be related to the pro-convulsive effect of tramadol at 100 mg/kg.

PCP-induced increases in locomotor activity and repetitive movements, observed at high doses [17], are possibly related to NMDA blockade [44], the stimulation of dopamine transmission [45,46,47,48] or the agonistic action at dopamine D2 receptors [47,48]. On the other hand, only the highest dose of PCP (10 mg/kg) induced a transient reduction in the performance on the rod, probably due to the appearance of stereotyped movements [17,49].

### 3.4. Effect on Core Body Thermoregulation

While the three stereoisomers did not affect the surface temperature (data not shown), they modified the core temperature [5]. We have previously demonstrated that ortho and meta derivatives produced a mild core hyperthermia at the lowest dose (0.1 mg/kg) and a transient hypothermia at the highest dose (100 mg/kg), and the meta derivate seems to be more effective than the ortho compound. Otherwise, the 1-cyclohexyl-para-methoxybenzene induced only transient hypothermia at the higher dose (100 mg/kg), similar to tramadol (earlier at 100 mg/kg) and PCP (later at 1 and 10 mg/kg). The derivatives that affect thermoregulation are insensitive to naloxone, suggesting that opioid receptors are not involved. In the literature, tramadol and PCP produced hyperthermia, hypothermia or a biphasic effects, depending upon the doses used [50] and the ambient temperatures [51]. Our experimental paradigm had a biphasic effect on tramadol and only a hypothermic effect on PCP. The present data are in line with the significant hypothermia caused by tramadol in mice [52]. The hypothermia induced by tramadol was partially prevented by naloxone, suggesting that other mechanisms come into play in the thermoregulation exerted by tramadol. Indeed, tramadol modulates the release of both NE and 5HT, either of which are directly involved in thermoregulation mechanisms [53]. On the other hand, PCP showed no significant alterations in body temperature in the 3 h after the injection, as reported by [54]. Since the effects of stereoisomers are naloxone-independent, we can speculate that ortho, meta and para could affect body temperature in mice through non-opioid mechanisms, and possibly through NE/5-HT-receptor mechanisms.

### 3.5. Effect on Acute Mechanical and Thermal Analgesia

In the tail pinch test, ortho and meta derivatives evoke a transient mechanical analgesic effect at the dose of 10 mg/kg, while tramadol and PCP demonstrated a larger and faster analgesic effect. In the tail-withdrawal test, the para compound sustains thermal analgesia up to a 100 mg/kg dose, while the ortho and meta derivatives show activity at 10 mg/kg but lose their activity at the highest dose (100 mg/kg; [5]). The acute analgesic effect induced by the derivatives is naloxone-insensitive, suggesting that opioid receptors are not involved in this action. Tramadol- and PCP-induced transient analgesia peaked in the first hour. Analgesia induced by tramadol was partially prevented by naloxone, while that induced by PCP was naloxone-insensitive.

A pharmacokinetic study on tramadol in B6 mice after IP administration at a dose of 25 mg/kg revealed that tramadol and its metabolite O-Dt reach similar plasma levels one hour after administration. The same study demonstrated that tramadol and O-Dt were quantifiable up to 4 and 2 h after administration, respectively [55]. These results reveal the high metabolism of tramadol compared to its metabolite O-Dt. Therefore, we suggest that the mechanical and thermal analgesic effects seen in the first 2 h of measurements after tramadol administration are mainly induced by O-Dt, which acts as a mu receptor agonist, eliciting naloxone-sensitive analgesic effects [56]. Interestingly, a recent structure–activity study of tramadol and its metabolite proved that O-Dt and morphine share common pharmacophore features with, and have similar binding modes to, the mu opioid receptor [57]. The experimental data suggest that tramadol and its metabolite, O-DT, exert their analgesic effect through the direct activation of mu opioid receptors, but also through the indirect activation of central α2-adrenoceptors [58,59] and descending serotonergic pathways [60]. Therefore, our data suggest that 1-cyclohexyl-x-methoxybenzene derivatives may share some antinociceptive mechanisms with tramadol and PCP, but not their opioidergic component. Further studies are needed to unveil the mechanisms responsible for the analgesic action of three stereoisomers.

### 3.6. Cardiorespiratory Effects

Ortho, meta and para increase breath rate without modifying SpO2, enhance heart rate and cause vasoconstriction, highlighting an action profile for the cardio-respiratory system similar to that of compounds stimulating the release of catecholamines, such as those recently reported for methiopropamine [42]. Their effects were naloxone-insensitive, suggesting that they are mediated by opioid-independent mechanisms. Conversely, tramadol and PCP reduce breath rate, SpO2 saturation and heart rate, although they use different mechanisms. Tramadol causes vasodilatation, while PCP causes vasoconstriction. Tramadol-induced cardio-respiratory changes were naloxone-sensitive, while those induced by PCP were not. In preclinical studies, a low dose of tramadol induced a slight increase in arterial blood pressure and heart rate in anaesthetized rabbits [61], dogs [62] and rats [63], possibly due to enhancement of the release of NE and/or 5-HT, which may result in peripheral vasoconstriction and/or heart stimulation [63]. While, at high doses, tramadol caused myocardial depression and hypotension in anaesthetized rabbits [64], dogs [65] and rats [66], possibly due to mu opioid receptor activation [66] and vascular relaxation as a result of nitric oxide production, and exerted a direct effect on smooth muscle [67]. These depressive effects are typically reported in human overdoses. In fact, the intravenous administration of tramadol caused orthostatic hypotension [68] and a transient rise in arterial blood pressure [69], while direct cardiotoxicity, characterized by cardiac arrest and severe biventricular failure, was a factor in intoxication [70] and deaths due to tramadol overdose [71,72]. Together with the cardio-depressive effect, tramadol causes respiratory depression in both animal models [73] and humans [11,18,74] possibly due to the stimulation of mu opioid receptors, which are reverted by naloxone. On the other hand, as previously reported, PCP reduced cardiorespiratory functions [17]. PCP has a complex pharmaco-dynamic profile on different receptor targets [75]; therefore, its cardiorespiratory action could be due to its interaction with the cardiac sigma-receptors [76], dopamine D2-receptors [77] and/or modulation of cardiac potassium channels [78]. Our results are consistent with the literature data showing the adverse effect of high doses of tramadol and PCP on cardiorespiratory functions. This outlines the differences observed with the 1-cyclohexyl-x-methoxybenzene derivatives that led to different modulation on the cardiorespiratory system, resembling the effect of stimulant drugs characterized by an increase in heart and breath rate, associated with vasoconstriction [42]. Further studies are needed to better understand the mechanisms underlying these cardiorespiratory effects that can predispose one to cardiac toxicity.

## 4. Materials and Methods

### 4.1. In Vitro Studies

#### 4.1.1. Drugs and Reagents

Brilliant black, bovine serum albumin (BSA), 4-(2-hydroxyethyl)-1-piperazineethanesulfonic acid (HEPES), and probenecid were from Sigma Aldrich (St. Louis, MO, USA). Pluronic acid and Fluo-4 AM were from Thermo Fisher Scientific (Waltham, MA, USA). All cells culture media and supplements were from Euroclone (Milano, Italy). Tramadol hydrochloride 50 mg/1 mL was purchased from Grünenthal Italia S.r.l and diluted in sterile water at a concentration of 10 mM, while its three analogs were dissolved in dimethyl sulfoxide (DMSO) at a concentration of 10 mM. The stock solutions were kept at −20 °C until use.

#### 4.1.2. Cells

CHO cells lines permanently co-expressing mu and kappa receptor with the C-terminally modified Gαqi5, and delta receptors with the C-terminally modified GαqG66Di5, were used. Details regarding the generation of these cells have been described previously [16,17]. Cells were cultured in culture medium consisting of Dulbecco’s modified Eagle’s medium (DMEM)/HAMS F12 (1:1), supplemented with 10% fetal bovine serum (FBS), penicillin (100 IU/mL), streptomycin (100 mg/mL), geneticin (G418; 200 µg/mL) and hygromycin B (100 µg/mL). Cell cultures were kept at 37 °C in 5% CO_2_/humidified air. When confluence was reached (3–4 days), cells were sub-cultured as required using trypsin/EDTA and used for experimentation. Cells were seeded at a density of 50,000 cells/well into 96-well, black, clear-bottom plates. After 24 h incubation, the cells were loaded with Hank’s Balanced Salt Solution (HBSS), supplemented with 2.5 mM probenecid, 3 µM of the calcium-sensitive fluorescent dye Fluo-4 AM, 0.01% pluronic acid and 20 mM HEPES (pH 7.4) for 30 min at 37 °C. Then, the loading solution was aspirated and a washing step with 100 µL/well of HBSS, HEPES (20 mM, pH 7.4), 2.5 mM probenecid and 500 µM Brilliant Black was carried out. Subsequently, 100 µL/well of the same buffer was added. After placing cell culture and compound plates into the FlexStation II (Molecular Devices, Sunnyvale, CA, USA), changes in the fluorescence of the cell-loaded, calcium-sensitive dye Fluor-4 AM were measured. On-line additions were carried out in a volume of 50 μL/well.

#### 4.1.3. Data Analysis and Terminology

All data were analyzed using Graph Pad Prism 6.0 (La Jolla, CA, USA). Data are expressed as mean ± sem of n experiments performed in duplicate. Agonist effects were expressed as maximum change in percent over the baseline fluorescence. Baseline fluorescence was measured in wells treated with vehicle. Agonist potency was expressed as pEC50, which is the negative logarithm to base 10 of the agonist molar concentration that produces 50% of the maximal possible effect of that agonist. The agonist concentration–response curves were fitted with the four-parameter logistic nonlinear regression model:

$$\mathrm{Effect}=\mathrm{Baseline}+\frac{\left(E_{\max}\;-\;\mathrm{Baseline}\right)}{\left(1+10^{({\mathrm{LogEC}}_{50}-{\mathrm{Log}}_{\left[\mathrm{compound}\right)}\mathrm{Hillslope}}\right)}$$

### 4.2. In Vivo Studies

#### 4.2.1. Animals

Four hundred and fifty-four male ICR (CD-1^®^) mice weighing 30–35 g (Centralized Preclinical Research Laboratory, University of Ferrara, Italy) were group housed (5 mice per cage; floor area per animal was 80 cm^2^; minimum enclosure height was 12 cm), exposed to a 12:12-h light–dark cycle (light period from 6:30 AM to 6:30 PM) at a temperature of 20–22 °C and humidity of 45–55%, and were provided with ad libitum access to food (Diet 4RF25 GLP; Mucedola, Settimo Milanese, Milan, Italy) and water. The experimental protocols performed in the present study were in accordance with the U.K. Animals (Scientific Procedures) Act of 1986 and associated guidelines and the new European Communities Council Directive of September 2010 (2010/63/EU). Experimental protocols were approved by the Italian Ministry of Health (license n. 335/2016-PR) and by the Animal Welfare Body of the University of Ferrara. According to the ARRIVE guidelines, all possible efforts were made to minimise the number of animals used, minimise the animals’ pain and discomfort and reduce the number of experimental subjects. For the overall study, 454 mice were used. In the battery of behavioral tests used in the safety pharmacology studies (see material and methods), for each treatment of (vehicle, 4 different 1-cyclohexyl-x-methoxybenzene derivatives or tramadol doses, 0.1, 1, 10 and 100 mg/kg), 8 mice were used (total mice used: 136). In the safety pharmacology studies, performed with naloxone, for each treatment of (naloxone 6 mg/kg, naloxone+1-cyclohexyl-x-methoxybenzene derivatives (100 mg/kg for each compound), naloxone+tramadol 100 mg/kg and naloxone+PCP 10 mg/kg), 6 mice were used (total mice used: 36). In the analysis of spontaneous locomotion in the open-field test, for each treatment of (vehicle or 4 different 1-cyclohexyl-x-methoxybenzene derivatives or tramadol doses, 0.1, 1, 10 and 100 mg/kg), 8 mice were used (total mice used: 160), while, for the antagonism studies with naloxone, for each treatment of (naloxone 6 mg/kg, naloxone+1-cyclohexyl-x-methoxybenzene derivatives (10 and 100 mg/kg for ortho and meta and 100 mg/kg para), naloxone+tramadol 1 and100 mg/kg and naloxone+PCP 10 mg/kg), 6 mice were used (total mice used: 54); in the cardiorespiratory studies, for each treatment of (vehicle, the highest 1-cyclohexyl-x-methoxybenzene derivatives (100 mg/kg) and tramadol (100 mg/kg), 6 mice were used (total mice used: 30) while for the antagonism studies with naloxone, for each treatment of (naloxone 6 mg/kg, naloxone+1-cyclohexyl-x-methoxybenzene derivatives (100 mg/kg for each compound), naloxone+tramadol 100 mg/kg and naloxone+PCP 10 mg/kg) 5 mice were used (total mice used: 30).

#### 4.2.2. Drug Preparation and Dose Selection

Tramadol hydrochloride ((±)cis-2-[(dimethylamino)methyl]-1-(3-methoxyphenyl) cyclohexanol hydrochloride) and phencyclidine hydrochloride (1-(1-phenylcyclohexyl) piperidine hydrochloride) were purchased from LGC standards (LGC Standards S.r.l., Sesto San Giovanni, Milan, Italy), while naloxone was purchased from Sigma Aldrich (St. Louis, MO, USA). 1-cyclohexyl-x-methoxybenzene derivatives (ortho, meta and para) were synthesized and purified as previously reported [5]. Drugs were initially dissolved in absolute ethanol (final concentration was 2%) and Tween 80 (2%) and brought to the final volume with saline (0.9% NaCl). The solution made with ethanol, Tween 80 and saline was also used as the vehicle. Drugs were administered by intraperitoneal injection at a volume of 4 ul/g. Doses of ortho, meta, para, tramadol (0.1–100 mg/kg i.p.) and PCP (0.01–100 mg/kg used in core temperature and naloxone studies) were chosen in previous studies [5,17]. To compare the effects of 1-cyclohexyl-x-methoxybenzene derivatives and tramadol with those of PCP (0.01–10 mg/kg), we carried out preliminary studies (*n* = 3 mice) evaluating the effect 1-cyclohexyl-x-methoxybenzene derivatives and tramadol at a dose of 0.01 mg/kg. Since this dose was ineffective in each experimental paradigm studied, in order to reduce the number of animals used (3-R rule), we did not carry out further experiments with the dose of 0.01 mg/kg, considering it completely ineffective. The dose–response effects of PCP were obtained by [17]. The dose of 100 mg/kg of PCP (for comparison with 1-cyclohexyl-x-methoxybenzene derivatives and tramadol at 100 mg/kg) was only tested in 3 mice, since it causes convulsion and death of the animal 5–15 min after administration. The high dose of naloxone (6 mg/kg) was used to achieve a complete blockade of the mu, delta and kappa opioid receptors [28].

### 4.3. Behavioral Studies

The effects of 1-cyclohexyl-x-methoxybenzene derivatives, tramadol and PCP were investigated using a battery of behavioural tests that are widely used in pharmacology safety studies for the preclinical characterization of new psychoactive substances in rodents [5,17,32,79,80,81]. All experiments were performed between 8:30 AM and 2:00 PM. Experiments were conducted blindly by trained observers working in pairs [82]. Mouse behaviour (sensorimotor responses) was videotaped and analysed offline by a different trained operator, who provided the test scores.

#### 4.3.1. Major Neurological Changes

Neurological changes in the mice, such as tail elevation, hyperreflexia and convulsions, were evaluated as previously described [79,80,81]. Neurological changes are expressed as frequency (percent of animals that develop symptoms), duration (total time in seconds), latency (time in seconds of symptom onset) and score (degree of tail elevation). Tail elevation was measured during the observation of freely moving mice in a square area (score 0/4 no tail elevation, score 4/4 Straub tail).

#### 4.3.2. Sensorimotor Studies

We studied the voluntary and involuntary sensorimotor responses of the mice, resulting from different reactions to visual, acoustic and tactile stimuli [80].

##### Evaluation of the Visual Response

Visual response was verified by two behavioral tests, which evaluated the ability of the animal to capture visual information when the animal was either stationary (the visual object response) or moving (the visual placing response).

Visual object response test was used to evaluate the ability of the mouse to see an object approaching from the front (frontal view) or the side (lateral view), which typically induces the animal to shift or turn the head, bring the forelimbs in the position of “defense” or retreat. For the frontal visual response, a white horizontal bar was moved frontally to the mouse head, and the manoeuvre was repeated three times. For the lateral visual response, a small dentist’s mirror was moved into the mouse’s field of view in a horizontal arc, until the stimulus was between the mouse’s eyes. The procedure was conducted bilaterally [17,80] and repeated three times. The assigned score was 1 if there was a reflection in the mouse movement, or 0 if it was not present. The total value was calculated by adding the scores obtained in the frontal to those obtained in the lateral visual object response test (overall score: 9). The visual object response was measured at 0, 10, 30, 60, 120, 180, 240 and 300 min post-injection.

The Visual Placing response test was performed using a tail suspension modified apparatus, able to bring the mouse towards the floor at a constant speed of 10 cm/sec [80]. In brief, CD-1 mice were suspended 20 cm above the floor by an adhesive tape, placed approximately 1 cm from the tip of the tail. The downward movement of the mouse was videotaped by a camera (B/W USB Camera, day and night, with varifocal lens; Ugo Basile, Gemonio, VA, Italy) placed at the base of the tail suspension apparatus. The films were analyzed off-line by a trained operator who was unaware of the drug treatments performed. The frame-by-frame analysis allowed for an evaluation of the beginning of the mouse’s reaction, while it was approaching the floor. The first movement of the mouse when it perceives the floor is the extension of the front legs. When the mouse started the reaction, an electronic ruler evaluated the perpendicular distance in millimeters between the eyes of the mice and the floor. Untreated control mice typically perceive the floor and prepare to contact at a distance of about 25 ± 4.8 mm. The visual placing response was measured at 0, 15, 40, 70, 130, 190, 250 and 310 min post-injection.

##### Evaluation of Acoustic Response

Acoustic response measures the reflexes of the mouse in response to an acoustic stimulus produced behind the animal. In particular, four acoustic stimuli of different intensities and frequencies were tested [80]. Each sound test was repeated three times. A score of 1 was given if there was a response and a score of 0 was given if there was no response, for a total score of 3 for each sound. The acoustic total score was calculated by adding scores obtained in the four tests (overall score of 12). The acoustic response was measured at 0, 10, 30, 60, 120, 180, 240 and 300 min post-injection.

##### Evaluation of Core Body Temperature

The core temperature was determined using a probe (1 mm diameter) that was gently inserted, after lubrication with liquid Vaseline, into the rectum of the mouse (to about 2 cm), and left in position until the temperature stabilised (about 10 s; [79]). The probe was connected to a Cole Parmer digital thermometer, model 8402. Core body temperature was measured at 0, 30, 50, 85, 140, 200, 260 and 320 min post-injection.

##### Evaluation of Pain Induced by a Mechanical and a Thermal Stimulus

Acute mechanical nociception was evaluated using the tail pinch test [79]. A special rigid probe connected to a digital dynamometer (ZP-50N, IMADA, Japan) was gently placed on the tail of the mouse (in the distal portion), and progressive pressure was applied. When the mouse flicked its tail, the pressure was stopped and the digital instrument recorded the maximum peak of weight that was supported (g/force). A cut-off (500 g/force) was set to avoid tissue damage. The test was repeated three times, and the final value was calculated by averaging the three obtained scores. Acute thermal nociception was evaluated using the tail withdrawal test [79]. The mouse was restrained in a dark plastic cylinder and half of its tail was dipped in 48 °C water, Then, the length of time (in s) for which the tail was left in the water was recorded. A cut-off (15 s) was set to avoid tissue damage. Acute mechanical and thermal nociception was measured at 0, 35, 55, 90, 145, 205, 265 and 325 min post-injection.

##### Motor Activity Assessment

Alterations in motor activity were measured using the bar, the accelerod tests and the analysis of spontaneous locomotor activity [28,79]. In the bar test, the mouse’s forelimbs were placed on a plastic bar (height 6 cm). The time spent on the bar was measured (immobility cut off: 20 s), and akinesia was calculated as the total time spent on the bar after three consecutive trials (total maximal time of catalepsy: 60 s). The bar test was performed at 0, 20, 40, 70, 140, and 195 min post-injection. In the accelerod test, the animals were placed on a rotating cylinder that automatically increases in velocity in a constant manner (0–60 rotations/min in 5 min). The time spent on the cylinder was measured. The accelerod test was performed at 0, 40, 60, 95, 150, 210, 270 and 330 min post-injection. Spontaneous locomotor activity was measured using the ANY-maze video-tracking system (Ugo Basile, application version 4.99 g Beta). The mouse was placed in a square plastic cage (60 × 60 cm) located in a sound- and light-attenuated room, and motor activity was monitored for 240 min. Four mice were monitored at the same time in each experiment. The spontaneous locomotor activity is measured as the horizontal distance travelled in meters (m). The distance travelled was analysed every 15 min, for a maximum of 240 min. To avoid mice olfactory cues, cages were carefully cleaned with a diluted (5%) ethanol solution and washed with water between animal trials. All experiments were performed between 9:00 AM and 1:00 PM.

### 4.4. Cardiorespiratory Analysis

To monitor cardiorespiratory parameters in awake and freely moving mice with no invasive instruments and minimal handling, a collar with a sensor was applied to continuously detect breath rate, oxygen saturation, heart rate and pulse distension (vessel diameter changes), with a frequency of 15 Hz [28,83]. During the experiment, the mouse was allowed to freely move in a cage (40 × 40 × 30 cm) with no access to food and water while being monitored by the sensor collar through MouseOx Plus (STARR Life Sciences^®^ Corp. Oakmont, PA, USA) software. In the first hour of acclimation, a fake collar, similar to the real one used in the test but with no sensor, was used to minimize the potential stress during the experiment. Then, the real collar (with sensor) was replaced, and baseline parameters were monitored for 60 min. Subsequently, 1-cyclohexyl-x-methoxybenzene derivatives, tramadol (100 mg/kg), PCP (10 mg/kg) or vehicle were administered, and data were recorded for up to 240 min. Cardiorespiratory changes were analysed and reported at 0, 15, 30, 60, 120, 180 and 240 min post-injection.

### 4.5. Data and Statistical Analysis

Core temperature values are expressed as the difference between control temperature (before injection) and temperature following drug administration (Δ°C). Antinociception (tail withdrawal and tail pinch tests) and catalepsy (bar test) are calculated as the percent of maximal possible effect {EMax% = ((test-control latency)/(cut off time-control)) × 100}. Data are expressed in absolute values (seconds (sec) in neurological changes, meters (m) for distance travelled), Δ°C (core and surface temperature), Emax% (tail withdrawal, tail pinch and bar test) and percentage of basal (accelerod test). In sensorimotor response experiments, data are expressed in arbitrary units (visual objects response and acoustic response) or percentage of baseline (visual placing response). Data are expressed in percentage of basal value (respiratory rate (expressed as respiratory rate per minute (rrpm), SpO2 saturation (oxygen blood saturation expressed as %), heart rate (expressed as heart beats per min (bpm) and pulse distention (vessel diameter changes expressed as µm)). All the numerical data are given as mean ± SEM of 4 independent experimental replications. The statistical analysis of the effects of the individual substances in different concentrations over time, and analysis of the antagonism studies, were performed using a two-way ANOVA, followed by a Bonferroni test for multiple comparisons. The statistical analysis of comparisons of the maximal effects induced by treatments were analyzed by one-way ANOVA, followed by a Bonferroni test for multiple comparisons. Student’s *t*-test was used to determine statistical significance (*p* < 0.05) between two groups (see neurological changes). The statistical analysis was performed using Prism software (GraphPad Prism, San Diego, CA, USA). All analyses were performed using GraphPad Prism software.

## 5. Conclusions

The present study demonstrates in vitro that the three 1-cyclohexyl-x-methoxybenzene stereoisomers, similar to tramadol, were inactive at mu, kappa and delta receptors. Therefore, their opioid-dependent in vivo effects, such as impairments of visual sensorimotor responses, may be due to metabolic activation, as reported for the O-Dt metabolite of tramadol [25]. Further studies will be undertaken to verify this possibility.

In vivo studies show that three stereoisomers may induce pharmaco-toxicological effects that resemble, in some experimental paradigms, those induced by tramadol and PCP. Notably, they could alter sensorimotor visual responses though mechanisms involving the opioid system and affect, in a naloxone-independent manner, thermoregulation, mechanical and thermal pain threshold, motor activity, and cardiorespiratory functions, highlighting their potentially dangerous effects.

Therefore, further studies on the receptor activity of the 1-cyclohexyl-x-methoxybenzene are recommended, in order to better understand their potential for abuse and the dangers they pose in many daily activities, greatly increasing the risk factors for workplace accidents and traffic injuries.

## Figures and Tables

**Table 1:** Potencies (pEC50) and maximal effects of tramadol and 1-cyclohexyl-x-methoxybenzene derivatives at mu, kappa and delta opioid receptors. Data are means of 3–4 separate experiments performed in duplicate.

	Mu		Kappa		Delta	
Compounds	pEC_50_ (CL_95%_)	E_max_ ± sem %	pEC_50_ (CL_95%_)	E_max_ ± sem %	pEC50 (CL95%)	E_max_ ± sem %
***Standard agonists***	7.76 (7.53–7.99)	295 ± 33%	8.50 (8.32–8.68)	189 ± 16%	7.65 (7.36–7.94)	248 ± 19%
***Tramadol***	Inactive		Inactive		Crc incomplete, at 100 mM 102 ± 9%	
***Ortho***	Inactive		Inactive		Crc incomplete, at 100 mM 115 ± 7%	
***Meta***	Inactive		Inactive		Crc incomplete, at 100 mM 115 ± 7%	
***Para***	Inactive		Inactive		Crc incomplete, at 100 mM 97 ± 8%	

The standard agonists for mu, kappa and delta receptors were Dermorphin, Dynorphin A and DPDPE, respectively.

**Table 2:** Summary of the most important effects induced by the molecules, tested using neurological, behavioral and cardiorespiratory tests.

	1-Cyclohexyl-x-Methoxybenzene (0.1–100 mg/kg i.p)			Tramadol (0.1–100 mg/kg i.p)	PCP (0.01–10 mg/kg i.p)
	*ortho*	*meta*	*para*		
**Neurological changes**	no effect			tail elevation and convulsion NLX insensitive	no effect
**Visual object response**	dose-dependent inhibition NLX partially sensitive			dose-dependent inhibition NLX partially sensitive	dose-dependent inhibition NLX insensitive
**Visual placing response**	dose-dependent inhibition NLX partially sensitive	dose-dependent inhibition NLX partially sensitive	dose-dependent inhibition NLX sensitive	dose-dependent inhibition NLX sensitive	dose-dependant inhibition NLX insensitive
**Acoustic** **response**	dose-dependent inhibition NLX insensitive			dose-dependent inhibition NLX insensitive	dose-dependent inhibition NLX insensitive
**Core body** **temperature**	dose-dependent inhibition NLX insensitive			dose-dependent inhibition NLX partially sensitive	dose-dependent inhibition NLX insensitive
**Mechanical** **analgesia**	mild analgesia NLX insensitive	mild analgesia NLX insensitive	no effect	dose-dependent analgesia NLX partially sensitive	dose-dependent analgesia NLX insensitive
**Thermal** **analgesia**	dose-dependent inhibition NLX insensitive			dose-dependent analgesia NLX partially sensitive	dose-dependent analgesia NLX insensitive
**Bar test**	no effect			no effect	no effect
**Stimulated** **locomotion**	no effect			biphasic effect NLX insensitive	biphasic effect NLX insensitive
**Sponotaneous locomotion**	biphasic effect NLX insensitive	biphasic effect NLX insensitive	inhibitory effect NLX insensitive	biphasic effect NLX partially sensitive with low dosage	biphasic effect NLX insensitive
**Breath rate**	increased NLX insensitive	increased NLX insensitive	no effect	decreased NLX sensitive	decreased NLX insensitive
**SpO2** **saturation**	no effect	no effect	no effect	decreased NLX sensitive	decreased NLX insensitive
**Heart rate**	increased NLX insensitive	increased NLX insensitive	increased NLX insensitive	decreased NLX sensitive	decreased NLX insensitive
**Pulse** **distention**	decreased NLX sensitive	decreased NLX sensitive	decreased NLX sensitive	increased NLX sensitive	decreased NLX insensitive

## Data Availability

The data presented in this study are available on request from the first (S.B.) and corresponding author (M.M.) for researchers of academic institutes who meet the criteria for access to the confidential data.
